# Concentrations of Plasma Free Palmitoleic and Dihomo-Gamma Linoleic Fatty Acids Are Higher in Children with Abdominal Obesity

**DOI:** 10.3390/nu10010031

**Published:** 2018-01-01

**Authors:** Juan C. Aristizabal, Laura I. González-Zapata, Alejandro Estrada-Restrepo, Julia Monsalve-Alvarez, Sandra L. Restrepo-Mesa, Diego Gaitán

**Affiliations:** 1 Grupo de Investigación en Fisiología y Bioquímica (PHYSIS), Universidad de Antioquia, Medellín 050010, Colombia; 2 Escuela de Nutrición y Dietética, Universidad de Antioquia, Medellín 050025, Colombia; laura.gonzalez@udea.edu.co (L.I.G.-Z.); alejandro.estrada@udea.edu.co (A.E.-R.); julia.monsalve@udea.edu.co (J.M.-A.); 3 Grupo de Investigación en Determinantes Sociales y Económicos de la Salud y la Nutrición, Universidad de Antioquia, Medellín 050010, Colombia; 4 Grupo de Investigación en Demografía y Salud, Universidad de Antioquia, Medellín 050010, Colombia; 5 Grupo de Investigación Alimentación y Nutrición Humana, Universidad de Antioquia, Medellín 050010, Colombia; sandra.restrepo@udea.edu.co (S.L.R.-M.); diego.gaitan@udea.edu.co (D.G.)

**Keywords:** plasma free fatty acids profile, central obesity, waist circumference, cardiometabolic risk factors, gas chromatography

## Abstract

Increased plasma free fatty acids (FFAs) are associated with cardiometabolic risk factors in adults with abdominal obesity (AO). However, this association remains controversial in children. This study analyzed plasma FFA concentration in children with and without AO. Twenty-nine children classified with AO were matched by age and sex with 29 non-obese individuals. Blood samples were collected after fasting for 10–12 h. Plasma concentration of glucose, insulin, triglycerides, total cholesterol, low-density lipoprotein cholesterol (LDL-C), and high-density lipoprotein cholesterol (HDL-C) were determined by automatized methods. FFAs were analyzed by gas chromatography. Children with and without AO had similar age (7.1 ± 2.6 vs. 7.2 ± 2.7 years; *p* > 0.05) but obese children showed higher (*p* < 0.05) body mass index (BMI) (+4.3 kg/m^2^), systolic blood pressure (+5.1 mmHg), and insulin (+27.8 pmol/L). There were no significant differences in plasma total FFA concentration between groups (1.02 ± 0.61 vs. 0.89 ± 0.37 mmol/L; *p* > 0.05). However, children with AO had higher palmitoleic acid (0.94 vs. 0.70 wt %; *p* < 0.05) and dihomo-gamma linoleic acid (DHGL) (2.76 vs. 2.07 wt %; *p* < 0.05). Palmitoleic and DHGL acids correlated (*p* < 0.05) with BMI (*r* = 0.397; *r* = 0.296, respectively) and with waist circumference (*r* = 0.380; *r* = 0.276, respectively). Palmitoleic acid correlated positively with systolic blood pressure (*r* = 0.386; *p* < 0.05) and negatively with HDL-C (−0.572; *p* < 0.01). In summary, children with AO have higher plasmatic concentrations of free palmitoleic and DHGL fatty acids, which correlate with cardiometabolic risk factors.

## 1. Introduction

Abdominal obesity (AO) represents an increase in visceral and subcutaneous adipose tissue, and it is associated with excessive chronic energy intake and a sedentary lifestyle [1,2,3]. AO is a cardiovascular risk factor and a major predictor of the metabolic abnormalities observed in the metabolic syndrome (e.g., dyslipidemia, high blood pressure, and impaired glucose metabolism) [4,5]. Circulating concentrations of free fatty acids (FFAs) have been proposed to be a possible link between abdominal obesity and cardiometabolic alterations [6,7]. According to the “fatty acids theory”, hypertrophied insulin resistant adipocytes release excessive fatty acids (e.g., FFAs) to blood circulation. This promotes ectopic lipid accumulation and insulin resistance in key organs (e.g., liver, pancreas, heart, and skeletal muscle) and results in cardiometabolic alterations [7,8]. Data in adults show associations of plasma FFAs with insulin resistance, metabolic syndrome, increased risk of type-2 diabetes, and other obesity-related metabolic disorders [9,10,11,12]. Despite the fact that childhood obesity can trigger early development of cardiovascular diseases [13,14], there is limited research about the relationship between FFAs and cardiometabolic risk factors in pediatric populations [15,16]. 

Studies comparing plasma FFA concentration in children with and without obesity show mixed results. Sabin et al. [17] and Reinehr et al. [18] found higher concentrations of total FFAs in obese children compared to normal weight peers. In contrast, Chu et al. [19] and Bermudez et al. [20] did not find significant differences in total FFAs in obese children compared with normal weight kids; and Gil-Campos et al. [21] reported lower total FFAs in obese children. Several reasons may explain the contradictory results among studies, such as the inclusion of children at different pubertal stages, the lack of control by age and gender in the analysis, and the use of the body mass index (BMI) to classify obesity, since there is a high variability in the fat mass content for a given BMI in youth [22,23]. Recently (2014), the Identification and prevention of dietary- and lifestyle-induced health effects in children and infants-Study (IDEFICS) released waist circumference cut-offs obtained from a representative sample from nine European countries [24]. These reference values derived from normal-weight children offer a good option to classify AO in children [24].

Most studies in children only report the total FFA concentration [18,19,25,26,27]. However, the FFA profile adds significant information given that different fatty acids might have diverse metabolic effects [6,28]. The analysis of the FFA profile also allows to calculate the product–precursor ratio of several fatty acids, such as palmitoleic/palmitic, dihomo-gamma-linoleic/linoleic, and arachidonic/dihomo-gamma-linoleic, which are used to estimate delta-9, delta-5, and delta-6 desaturase activities, respectively [9,29]. Studies in adults showed that high activities of delta-9 and delta-6, and low activity of delta-5, are related to insulin resistance and metabolic syndrome [9,29,30,31].

A better understanding of the role of FFAs in the relationship between AO and cardiovascular risk factors may help to improve obesity prevention and treatment, an urgent task given the high worldwide prevalence of childhood obesity. Studies in children may provide key information since factors that affect FFA profile, such as smoking, alcohol intake, and medicament use, are better controlled. Furthermore, at young ages the long-term effects of obesity are not fully established. This study compared the FFA profile with estimated desaturase activities in children with and without AO, and examined how FFAs and desaturase activities are associated with cardiovascular risk factors and metabolic syndrome components. 

## 2. Materials and Methods 

This is a cross-sectional analytical study. A convenient subsample of 58 children from Medellin-Colombia were selected from the South American Youth/Child Cardiovascular and Environmental study (SAYCARE). SAYCARE is a running multicenter study in children and adolescents attending private and public schools at seven South American cities. SAYCARE aims to develop methods to collect reliable, comparable, and validated data about cardiovascular health biomarkers, lifestyles, and environmental, social, and familial factors. Children who were sick or under treatment with steroids or other kinds of hormones/medications were excluded. Twenty-nine abdominal obese children (17 boys and 12 girls) were matched one by one according to age and sex with non-abdominal obese peers. The group sample size was calculated with a power of 85% (at 95% level of confidence), which allowed for the detection of a minimum difference between groups of 4.1 μmol/L of myristic fatty acid, which has been previously reported in children [17]. The study was performed according to the Helsinki Declaration and was approved by the Bioethical Review Board of the Research System from the University of Antioquia, Medellín-Colombia (certificate number 14-43-596, 28 May 2014). Informed consent was obtained from all participants and guardians of the children.

Abdominal obesity assessed by waist circumference, and the other metabolic syndrome components (e.g., high blood pressure, low HDL-C concentrations, high triglyceride concentrations, and glucose intolerance or insulin resistance) were evaluated using as a cut-off the 90th-percentile reference value of the IDEFICS study [24,32,33].

Children’s parents/caregivers answered a semi-quantitative food frequency questionnaire (FFQ) in an oral interview with registered dietitians. The FFQ was designed with 70 items, each one with several answer options to capture the habitual food intake of the SAYCARE study participants. The FFQ was designed to collect information of food intake related to cardiovascular diseases, and was validated with multiple 24 h dietary recalls. The FFQ data were analyzed to estimate daily energy and macronutrient intake using the Colombian food database as described by others [34,35]. Due to food database limitations, the analysis provided total grams of saturated, monounsaturated, and polyunsaturated fat intake without specifying individual fatty acids.

Body weight was measured to the nearest 0.1 kg using a digital scale (WISO W801, Barreiros, Brazil). Height was measured to the nearest 0.1 cm using a stadiometer (Cardiomed WSC, Paraná, Brazil). Waist circumference was measured to the nearest 0.1 cm, midway between the lowest rib margin and the iliac crest, using a flexible tape (Cardiomed WSC, Paraná, Brazil). Biceps, triceps, subscapular, and iliocrestal skinfold thickness were measured on the right side of the body to the nearest millimeter, using a caliper (Lange, CA, USA) as described by Lohman [36]. Triceps and subscapular skinfolds were summed and the percentage of fat mass was calculated with Slaughter’s equation [37].

Participants were instructed to fast overnight for 10 to 12 h. Blood was drawn from the antecubital vein in ethylenediaminetetraacetic acid tubes. Blood was immediately centrifuged at 1500× *g* for 15 min at 4 °C. Plasma was aliquoted and kept frozen at −80 °C for further analysis. Plasma glucose and lipid profile were measured by colorimetric and enzymatic methods using an automatic analyzer (Roche, Cobas c501, Mannheim, Germany). Insulin was measured by a chemiluminescence method using an automatic analyzer (Roche, Cobas c501, Mannheim, Germany). High-sensitive C-reactive protein (hs-CRP) was measured by a turbidimetric method using an automatic analyzer (Roche, Cobas c501, Mannheim, Germany). The coefficients of variation for these essays were as follows: glucose: 1.1%; insulin: 4.2%; triglycerides: 1.3%; total cholesterol: 1.2%; HDL-C: 1.6%; hs-CRP: 1.8%. HOMA-IR was calculated as plasma glucose (mmol/L) × plasma insulin (mU/L)/22.5 [38]. 

Plasma lipids were extracted by the Folch method [39]. The free fatty acid fraction was separated from plasma lipids using solid-phase extraction (SPE) by column chromatography (SPE NH_2_ 300 mg, Sigma-Aldrich, St. Louis, MO, USA). Fatty acids were methylated with boron trifluoride (20% BF3-MeOH). The fatty acid methyl esters (FAMEs) were analyzed using gas chromatography (Agilent Technologies 7890B GC system, Santa Clara, CA, USA). The FAMEs were identified by comparison with authentic FAME standards and peak areas were integrated as relative weight (wt %) using OpenLab CDS ChemStation software (Agilent Technologies, Santa Clara, CA, USA). The coefficient of variation for the free fatty acid analysis was 1.3%. The product–precursor ratio of the FFAs palmitoleic (16:1, *n*-7)/palmitic (16:0), dihomo-gamma-linoleic (20:3 *n*-6)/linoleic (18:2 *n*-6), and arachidonic (20:4 *n*-6)/dihomo-gamma-linoleic (20:3 *n*-6) were used to estimate adipocytes’ activities of delta-9 desaturase, delta-6 desaturase, and delta-5 desaturase, respectively, as described by others [40,41]. 

Normal distribution of data was tested with the Kolmogorov–Smirnov test. Results are presented as means ± standard deviation or median and interquartile range according to the normal distribution of data. For normally distributed data, a *t*-test was used to test differences between non-obese and abdominal obese children; one-way ANOVA with Scheffé post-hoc was used to test differences between FFA concentrations, estimated desaturase activities and the metabolic syndrome components. For non-normally distributed data, Mann–Whitney and Kruskal–Wallis tests were used to compare two or more groups, respectively. Partial correlations adjusted by sex and age were run between FFA concentrations, estimated desaturase activities, and cardiometabolic factors. A *p*-value ≤ 0.05 was considered statistically significant.

## 3. Results

Fifty-eight children (29 with AO and 29 without AO; 3 to 10 years) were included in the study. There were 17 boys and 12 girls in each group, with a similar average age (7.1 ± 2.6 vs. 7.2 ± 2.7 years; *p* = 0.876) (Table 1).

There were no significant differences in energy or macronutrient intake between groups with and without AO. As expected, abdominal obese children compared with non-obese peers showed higher (*p* < 0.001) BMI (+4.3 kg/m^2^), waist circumference (+11.2 cm), skinfold thickness sum (+34.0 mm), and body fat percentage (+9.7%). In addition, the obese group had higher systolic blood pressure (+5.1 mmHg; *p* = 0.001), fasting insulin (+27.8 pmol/L; *p* = 0.032), and high sensitive C-reactive protein (hs-CRP +5.6 nmol/L; *p* = 0.016) (Table 1).

The FFA profile and estimated desaturase activities are presented in Table 2. Abdominal obese children showed a higher concentration of palmitoleic acid (+0.24 wt %; *p* = 0.038) and DHGL acid (+0.69 wt %; *p* = 0.015). Likewise, the obese group had higher estimated activity of delta-9 desaturase (+0.01; *p* = 0.010) and delta-6 desaturase (+0.04; *p* = 0.010) and a lower activity of delta-5 desaturase (−0.94; *p* = 0.010) (Table 2).

Selected correlation coefficients of anthropometric and cardiometabolic variables with FFAs and desaturase activities are presented in Table 3. Waist circumference and BMI showed similar patterns; they correlated positively with palmitoleic acid, DHGL acid, delta-9 desaturase, and delta-6 desaturase; conversely, they correlated negatively with delta-5 desaturase (Table 3). HDL-C correlated negatively with palmitoleic acid (*r* = −0.572; *p* < 0.01) and delta-9 desaturase (*r* = −0.540; *p* < 0.01). Systolic blood pressure showed weak but significant correlations with palmitic, palmitoleic, and DHGL acids, and it correlated with delta-9, delta-6, and delta-5 desaturases. Other weak correlations of fat mass percentage and insulin with FFAs and desaturases are shown in Table 3.

**Figure 1:**
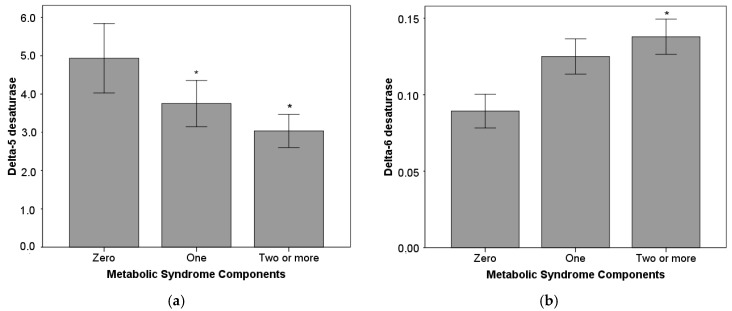
Estimated activities of delta-5 desaturase (**a**) and delta-6 desaturase (**b**) according to the metabolic syndrome components. The Kruskal–Wallis test for differences of Delta-5 desaturase and Delta-6 desaturase by metabolic syndrome components, with the Mann–Whitney Test for multiple comparison. * Different from zero components *p* < 0.05.

Estimated delta-6 and delta-5 desaturase activities were related to the metabolic syndrome components (Figure 1). Children who had one or more metabolic syndrome components showed lower delta-5 desaturase activity. In addition, children who had two or more metabolic syndrome components showed higher delta-6 desaturase activity (Figure 1).

## 4. Discussion

This study compared plasma FFA concentration with estimated desaturase activities in children with and without AO, and explored the associations between FFAs and cardiometabolic risk factors. Although no differences were found in total FFA concentration between groups, abdominal obese children showed differences in the plasma free fatty acid profile and estimated desaturase activities. Furthermore, some plasma FFAs correlated with cardiometabolic risk factors, suggesting that AO affects the FFA profile even at young ages.

Plasma total FFA concentrations did not differ between children with and without AO, and the groups’ values were similar to those reported in the literature between 0.332 and 1.02 mmol/L [17,25,27]. According to the “fatty acid theory”, obesity induces insulin resistance in adipocytes, triggering higher lipolysis rate and increased fatty acid release; these alterations augment FFA concentration in blood [7,8]. Several reasons may exist to explain the lack of higher total FFA concentration in obese kids: (a) abdominal obese children may have a normal insulin-response with respect to inhibition of lipolysis, as it has been described in obese adults [42,43]; (b) the elevated insulin concentration in obese kids (median = 70.2 pmol/L) may have prevented a raise in adipose tissue lipolysis; and (c) probably, children with AO did not present insulin resistance at this early age (3 to 10 years), given that their HOMA value (2.2) is still lower than the cut-off for insulin resistant (≥3.1). It is important to note that, in adolescents and adults, significant and positive associations have been observed between abdominal obesity, insulin resistance, and FFA concentrations [8,15,20,44].

Obese children showed higher concentrations of plasma non-esterified palmitoleic fatty acid. Palmitoleic acid mainly originates from de novo lipogenesis that occurs primarily in the liver and secondarily in the adipose tissue where this fatty acid is stored [45,46]. The higher concentration of palmitoleic acid in obese children may be due to a larger deposit in adipocytes, given that adipose tissue lipolysis is the major source of plasma FFAs at fasting state [40]. This idea is supported by the findings of Gong et al. [47], who reported higher concentrations of palmitoleic acid in adipose tissue of obese adults. Furthermore, in this study, obese children showed higher estimated delta-9 desaturase activity and fasting insulin concentration. Delta-9 desaturase originates palmitoleic acid by desaturation of palmitic acid in a key regulatory step of lipogenesis, a process highly stimulated by insulin and carbohydrate intake [45]. Nevertheless, no significant differences in carbohydrate intake were found between obese and non-obese children, probably due to inaccuracy of the FFQ [48]. In adults, in several lipids fractions, palmitoleic acid and delta-9 desaturase activity have been associated with increased risk of obesity, dyslipidemia, and insulin resistance [11,49,50,51,52]. This study found positive correlations of palmitic acid and delta-9 estimated desaturase activity with BMI, waist circumference, and systolic blood pressure, and negative correlations with HDL-C (Table 3).

Plasma non-esterified DHGL fatty acid concentrations were higher in obese children, and a similar result was reported in a previous study [20]. This is probably due to the endogenous metabolism of DHGL acid, since there were no differences in dietary PUFA intake, and DHGL acid is derived from the essential linoleic fatty acid [52]. Furthermore, the estimated activity of delta-6 desaturase—the rate-limiting enzyme in the production of DHGL from linoleic acid—was higher in obese children, probably stimulated by the hyperinsulinemia. Similar to a study evaluating obese adolescents, DHGL fatty acid was significantly correlated with BMI and waist circumference [20], and delta-6 desaturase activity was positively associated with the number of metabolic syndrome components (Figure 1).

In obese children, the plasma FFA ratio of 20:3 *n-*6/18:2 *n-*6 was higher and that of 20:4 *n-*6/20:3 *n-*6 was lower. These FFA ratios have been suggested to reflect delta-6 and delta-5 desaturase activities in adipocytes [40,41]. As mentioned before, the higher estimated delta-6 activity in obese children is probably due to higher insulin values, but delta-5 desaturase may not respond so strongly to environmental effects and may have a more genetic regulation [41]. Obese children showed low-grade systemic inflammation (median hs-CRP = 1.8 mg/L) and lower delta-5 desaturase activity, which could be part of a mechanism to avoid worsening the inflammatory state. Delta-5 desaturase uses DHGL acid to originate arachidonic acid (ARA), and these fatty acids are competitive substrates for the cyclooxygenase enzyme (COX) [52]. COX metabolites from DHGL acid are anti-inflammatory, and ARA metabolites are pro-inflammatory [52]. Therefore, keeping a low delta-5 activity in adipose tissue may help to downregulate ARA production and inflammation when the levels of DHGL are increased, as they are in obese kids. This theory could explain the negative correlation found between delta-5 desaturase activity and the number of metabolic syndrome components (Figure 1), given the close connection between low-grade inflammation and metabolic syndrome development.

To the best of our knowledge, this study is unique comparing plasma FFA concentration between children with and without AO paired by age and sex. An additional strength of this study is the use of specific sex and age cut-offs for waist circumference to classify AO. The IDEFICS references values showed to be a good option for this end, given that obese children showed higher systolic blood pressure, insulin concentrations, and hs-CRP levels and differed in the plasma FFA profile. The study has some limitations: the cross-sectional design does not permit determining causality. Moreover, the children age range (3–10 years) probably included kids with differences in habitual food intake and lifestyle. To control for these variables, and possible differences in pubertal status, the groups with and without AO were matched one by one according to age and sex. In addition, insulin resistance was estimated by HOMA; however, there are better methods like the oral glucose tolerance test and the intravenous glucose tolerance test. Similarly, waist circumference was used to establish AO, where techniques like the dual X-ray densitometry and magnetic resonance imaging are more precise. Nonetheless, we use waist circumference and HOMA because they provide acceptable information of AO and insulin resistance, respectively [5,53,54,55]. In addition, they are practical methods, easier to be accepted by the parents of our young participants than more invasive techniques. There are limitations for the FFQ application; for example, some foods might have been missed, since the FFQ relies on responders’ memories and some food items may not be listed in the instrument. The food database limitations did not allow for an estimated dietary intake of individual fatty acids in order to analyze how dietary fatty acids associate with the plasma FFA profile and the metabolic syndrome components.

## 5. Conclusions

Children with AO, compared to non-obese peers, showed higher systolic blood pressure, insulin concentrations, and hs-CRP levels, but no differences in total FFA concentration. However, abdominal obese kids had differences in the plasma FFA profile and estimated desaturase activities. Furthermore, some plasma FFAs were correlated with cardiometabolic risk factors, suggesting that AO affects the FFA profile even at young ages.

## Figures and Tables

**Table 1:** Characteristics of participants by group ^1^.

	Children without Abdominal Obesity (*n* = 29)	Children with Abdominal Obesity (*n* = 29)	*p*-Value
Age (years)	7.1 ± 2.6	7.2 ± 2.7	0.876
Energy intake (kcal/day)	2453 ± 946	2358 ± 781	0.704
Carbohydrate intake (g/day)	307.8 ± 134.6	278.6 ± 92.4	0.374
Protein intake (g/day)	84.9 ± 30.7	92.4 ± 42.3	0.478
Saturated fat intake (g/day)	37.8 ± 15.1	37.5 ± 12.8	0.942
Monounsaturated fat intake (g/day)	40.1 ± 17.8	40.2 ± 14.5	0.975
Polyunsaturated fat intake (g/day)	15.5 ± 8.4	14.1 ± 5.6	0.488
Body height (cm)	118.8 ± 16.8	122.4 ± 16.7	0.424
Body weight (kg)	22.1 ± 6.0	30.9 ± 10.7	0.000 *
Body mass index (kg/m^2^)	15.4 (1.5)	19.7 (3.5)	0.000 ^†^
Body mass index for age (z-score)	−0.2 ± 0.7	1.9 ± 0.8	0.000*
Waist circumference (cm)	53.7 (5.8)	64.9 (16.5)	0.000 ^†^
Skinfolds Sum (mm)	26.5 (9.8)	60.5 (36.8)	0.000 ^†^
Fat Percentage (%)	16.4 ± 5.5	26.1 ± 6.9	0.000 *
Systolic blood pressure (mmHg)	93.5 ± 6.4	98.6 ± 6.4	0.004 *
Diastolic blood pressure (mmHg)	58.5 ± 5.3	61.1 ± 6.5	0.096
Fasting blood glucose (mmol/L)	4.9 ± 0.3	4.8 ± 0.3	0.523
Fasting blood insulin (pmol/L)	42.4 (24.3)	70.2 (54.7)	0.032 ^†^
HOMA-IR	1.3 (0.7)	2.2 (1.8)	0.051
Triglycerides (mmol/L)	0.77 (0.53)	0.87 (0.45)	0.363
Total cholesterol (mmol/L)	4.12 (1.26)	3.97 (1.15)	0.363
HDL-C (mmol/L)	1.4 ± 0.3	1.3 ± 0.3	0.051
LDL-C (mmol/L)	2.3 ± 0.7	2.5 ± 0.7	0.246
Total FFAs (mmol/L)	1.02 (0.61)	0.89 (0.37)	0.258
Hs-CRP (nmol/L)	11.0 (7.8)	16.6 (14.2)	0.016 ^†^

^1^ Data presented as mean ± standard deviation or medians and interquartile range in parentheses according to the data distribution. * Differences between groups obtained with independent *t*-test. ^†^ Differences between groups obtained with the Mann–Whitney test. HOMA-IR: homeostasis model assessment of insulin resistance; HDL-C: high-density lipoprotein cholesterol; FFAs: free fatty acids; hs-CRP: high-sensitive C-reactive protein.

**Table 2:** Plasma free fatty acid proportions in children with and without abdominal obesity ^1^

	Children without Abdominal Obesity	Children with Abdominal Obesity	*p*-Value
Saturated fatty acids	42.77 ± 3.69	43.44 ± 3.63	0.485
Myristic (14:0) ^‡^	0.65 ± 0.23	0.67 ± 0.20	0.865
Palmitic (16:0)	26.32 ± 3.06	27.09 ± 3.39	0.365
Estearic (18:0)	16.02 ± 1.24	15.96 ± 1.24	0.858
Monounsaturated fatty acids	25.88 ± 11.30	25.26 ± 8.95	0.819
Palmitoleic (16:1 *n-*7) ^‡^	0.70 ± 0.29	0.94 ± 0.35	0.038 *
Oleic (18:1 *n-*9)	25.47 ± 11.33	24.71 ± 8.90	0.778
Polyunsaturated fatty acids	31.34 ± 8.00	31.28 ± 5.94	0.974
Linoleic (18:2 *n-*6)	19.72 ± 4.04	19.23 ± 3.97	0.603
DHGL (20:3 *n-*6)	2.07 ± 0.98	2.76 ± 1.09	0.015 *
Arachidonic (20:4 *n-*6)	6.75 ± 2.05	6.96 ± 2.35	0.729
DHA (22:6 *n-*3)	2.79 ± 1.80	2.34 ± 0.83	0.221
Fatty acids ratios			
16:1 n-7/16:0 (D9) ^‡^	0.03 ± 0.01	0.04 ± 0.01	0.010 *
20:3 n-6/18:2 *n-*6 (D6)	0.10 ± 0.04	0.14 ± 0.06	0.002 *
20:4 n-6/20:3 *n-*6 (D5)	3.47 (1.69)	2.53 (0.63)	0.002 ^†^
Omega-6/Omega-3	12.76 ± 4.55	13.64 ± 3.65	0.406

^1^ Data presented as mean ± standard deviation or medians and interquartile range in parentheses according to the data distribution. * Differences between groups obtained with independent *t*-test. ^†^ Differences between groups obtained with the Mann–Whitney test. **^‡^** Abdominal obese children *n* = 18, children without abdominal obesity *n* = 19. DHGL: dihomo-gamma-linoleic acid; DHA: docoxahexanoic acid; D9: delta-9 desaturase; D6: delta-6 desaturase; D5: delta-5 desaturase.

**Table 3:** Selected correlation coefficients of anthropometric and cardiometabolic variables with free fatty acids and estimated desaturase activities.

Anthropometric and Cardiometabolic	Palmitic (16:0)	Palmitoleic (16:1 *n-*7)	DHGL (20:3 *n*-6)	16:1 *n*-7/16:0 (D9)	20:3 *n*-6/18:2 *n*-6 (D6)	20:4 *n*-6/20:3 *n*-6 (D5)
Body mass index	0.145	0.397 *	0.296 *	0.451 *	0.342 *	−0.299 *
Waist circumference	0.160	0.380 *	0.276 *	0.414 *	0.332 *	−0.302 *
Fat mass percentage	0.146	0.338	0.232	0.403 *	0.289 *	−0.242
Systolic blood pressure	0.265 *	0.386 *	0.330 *	0.368 *	0.284 *	−0.314 *
Insulin	−0.014	0.272	0.132	0.306	0.142	−0.281 *
HDL-C	−0.164	−0.572 **	−0.198	−0.540 **	−0.250	0.242

Correlations in the whole group (*n* = 58) and adjusted by sex and age. * *p* < 0.05; ** *p* < 0.01. HDL-C: high-density lipoprotein cholesterol. DHGL: dihomo-gamma-linoleic acid; D9: delta-9 desaturase; D6: delta-6 desaturase; D5: delta-5 desaturase.

## References

[1] Smith U. (2015). Abdominal obesity: A marker of ectopic fat accumulation. J. Clin. Investig..

[2] Rosique-Esteban N., Diaz-Lopez A., Martinez-Gonzalez M.A., Corella D., Goday A., Martinez J.A., Romaguera D., Vioque J., Aros F., Garcia-Rios A. (2017). Leisure-time physical activity, sedentary behaviors, sleep, and cardiometabolic risk factors at baseline in the predimed-plus intervention trial: A cross-sectional analysis. PLoS ONE.

[3] Thorp A.A., McNaughton S.A., Owen N., Dunstan D.W. (2013). Independent and joint associations of tv viewing time and snack food consumption with the metabolic syndrome and its components; a cross-sectional study in australian adults. Int. J. Behav. Nutr. Phys. Act..

[4] Bays H.E., Toth P.P., Kris-Etherton P.M., Abate N., Aronne L.J., Brown W.V., Gonzalez-Campoy J.M., Jones S.R., Kumar R., La Forge R. (2013). Obesity, adiposity, and dyslipidemia: A consensus statement from the national lipid association. J. Clin. Lipidol..

[5] Bays H. (2014). Central obesity as a clinical marker of adiposopathy; increased visceral adiposity as a surrogate marker for global fat dysfunction. Curr. Opin. Endocrinol. Diabetes Obes..

[6] Sears B., Perry M. (2015). The role of fatty acids in insulin resistance. Lipids Health Dis..

[7] Grundy S.M. (2015). Adipose tissue and metabolic syndrome: Too much, too little or neither. Eur. J. Clin. Investig..

[8] Cooke A.A., Connaughton R.M., Lyons C.L., McMorrow A.M., Roche H.M. (2016). Fatty acids and chronic low grade inflammation associated with obesity and the metabolic syndrome. Eur. J. Pharmacol..

[9] Warensjö E., Rosell M., Hellenius M.L., Vessby B., De Faire U., Risérus U. (2009). Associations between estimated fatty acid desaturase activities in serum lipids and adipose tissue in humans: Links to obesity and insulin resistance. Lipids Health Dis..

[10] Stefan N., Stumvoll M., Bogardus C., Tataranni P.A. (2003). Elevated plasma nonesterified fatty acids are associated with deterioration of acute insulin response in igt but not ngt. Am. J. Physiol. Endocrinol. Metab..

[11] Paillard F., Catheline D., Duff F.L., Bouriel M., Deugnier Y., Pouchard M., Daubert J.C., Legrand P. (2008). Plasma palmitoleic acid, a product of stearoyl-coa desaturase activity, is an independent marker of triglyceridemia and abdominal adiposity. Nutr. Metab. Cardiovasc. Dis..

[12] Boden G. (2008). Obesity and free fatty acids. Endocrinol. Metab. Clin. N. Am..

[13] Juonala M., Viikari J.S., Raitakari O.T. (2013). Main findings from the prospective cardiovascular risk in young finns study. Curr. Opin. Lipidol..

[14] Juonala M., Magnussen C.G., Berenson G.S., Venn A., Burns T.L., Sabin M.A., Srinivasan S.R., Daniels S.R., Davis P.H., Chen W. (2011). Childhood adiposity, adult adiposity, and cardiovascular risk factors. N. Engl. J. Med..

[15] Toledo-Corral C.M., Alderete T.L., Richey J., Sequeira P., Goran M.I., Weigensberg M.J. (2015). Fasting, post-ogtt challenge, and nocturnal free fatty acids in prediabetic versus normal glucose tolerant overweight and obese latino adolescents. Acta Diabetol..

[16] Burrows T., Collins C.E., Garg M.L. (2011). Omega-3 index, obesity and insulin resistance in children. Int. J. Pediatr. Obes..

[17] Sabin M.A., De Hora M., Holly J.M., Hunt L.P., Ford A.L., Williams S.R., Baker J.S., Retallick C.J., Crowne E.C., Shield J.P. (2007). Fasting nonesterified fatty acid profiles in childhood and their relationship with adiposity, insulin sensitivity, and lipid levels. Pediatrics.

[18] Reinehr T., Kiess W., Andler W. (2005). Insulin sensitivity indices of glucose and free fatty acid metabolism in obese children and adolescents in relation to serum lipids. Metabolism.

[19] Chu N.F., Chang J.B., Shieh S.M. (2003). Plasma leptin, fatty acids, and tumor necrosis factor-receptor and insulin resistance in children. Obes. Res..

[20] Bermúdez-Cardona J., Velásquez-Rodríguez C. (2016). Profile of free fatty acids and fractions of phospholipids, cholesterol esters and triglycerides in serum of obese youth with and without metabolic syndrome. Nutrients.

[21] Gil-Campos M., del Carmen Ramírez-Tortosa M., Larqué E., Linde J., Aguilera C.M., Cañete R., Gil A. (2008). Metabolic syndrome affects fatty acid composition of plasma lipids in obese prepubertal children. Lipids.

[22] Freedman D.S., Wang J., Maynard L.M., Thornton J.C., Mei Z., Pierson R.N., Dietz W.H., Horlick M. (2005). Relation of bmi to fat and fat-free mass among children and adolescents. Int. J. Obes. (Lond.).

[23] Maynard L.M., Wisemandle W., Roche A.F., Chumlea W.C., Guo S.S., Siervogel R.M. (2001). Childhood body composition in relation to body mass index. Pediatrics.

[24] Nagy P., Kovacs E., Moreno L.A., Veidebaum T., Tornaritis M., Kourides Y., Siani A., Lauria F., Sioen I., Claessens M. (2014). Percentile reference values for anthropometric body composition indices in european children from the idefics study. Int. J. Obes. (Lond.).

[25] Garcés C., Cano B., Granizo J.J., Benavente M., Viturro E., Gutiérrez-Guisado J., de Oya I., Lasunción M.A., de Oya M. (2005). Insulin and homa in spanish prepubertal children: Relationship with lipid profile. Clin. Biochem..

[26] Salgin B., Ong K.K., Thankamony A., Emmett P., Wareham N.J., Dunger D.B. (2012). Higher fasting plasma free fatty acid levels are associated with lower insulin secretion in children and adults and a higher incidence of type 2 diabetes. J. Clin. Endocrinol. Metab..

[27] Hershkop K., Besor O., Santoro N., Pierpont B., Caprio S., Weiss R. (2016). Adipose insulin resistance in obese adolescents across the spectrum of glucose tolerance. J. Clin. Endocrinol. Metab..

[28] Pagadala M., Kasumov T., McCullough A.J., Zein N.N., Kirwan J.P. (2012). Role of ceramides in nonalcoholic fatty liver disease. Trends Endocrinol. Metab..

[29] Kawashima A., Sugawara S., Okita M., Akahane T., Fukui K., Hashiuchi M., Kataoka C., Tsukamoto I. (2009). Plasma fatty acid composition, estimated desaturase activities, and intakes of energy and nutrient in japanese men with abdominal obesity or metabolic syndrome. J. Nutr. Sci. Vitaminol. (Tokyo).

[30] Warensjö E., Risérus U., Vessby B. (2005). Fatty acid composition of serum lipids predicts the development of the metabolic syndrome in men. Diabetologia.

[31] Zhou Y.E., Egeland G.M., Meltzer S.J., Kubow S. (2009). The association of desaturase 9 and plasma fatty acid composition with insulin resistance-associated factors in female adolescents. Metabolism.

[32] De Henauw S., Michels N., Vyncke K., Hebestreit A., Russo P., Intemann T., Peplies J., Fraterman A., Eiben G., de Lorgeril M. (2014). Blood lipids among young children in europe: Results from the european idefics study. Int. J. Obes. (Lond.).

[33] Peplies J., Jiménez-Pavón D., Savva S.C., Buck C., Günther K., Fraterman A., Russo P., Iacoviello L., Veidebaum T., Tornaritis M. (2014). Percentiles of fasting serum insulin, glucose, hba1c and homa-ir in pre-pubertal normal weight european children from the idefics cohort. Int. J. Obes. (Lond.).

[34] Patel P.S., Sharp S.J., Jansen E., Luben R.N., Khaw K.T., Wareham N.J., Forouhi N.G. (2010). Fatty acids measured in plasma and erythrocyte-membrane phospholipids and derived by food-frequency questionnaire and the risk of new-onset type 2 diabetes: A pilot study in the european prospective investigation into cancer and nutrition (epic)-norfolk cohort. Am. J. Clin. Nutr..

[35] Bingham S.A., Gill C., Welch A., Cassidy A., Runswick S.A., Oakes S., Lubin R., Thurnham D.I., Key T.J., Roe L. (1997). Validation of dietary assessment methods in the uk arm of epic using weighed records, and 24-hour urinary nitrogen and potassium and serum vitamin c and carotenoids as biomarkers. Int. J. Epidemiol..

[36] Lohman T., Roche A., Martorell R. (1988). Antropometric Standardization Reference Manual.

[37] Slaughter M.H., Lohman T.G., Boileau R.A., Horswill C.A., Stillman R.J., Van Loan M.D., Bemben D.A. (1988). Skinfold equations for estimation of body fatness in children and youth. Hum. Biol..

[38] Wallace T.M., Matthews D.R. (2002). The assessment of insulin resistance in man. Diabet. Med..

[39] Folch J., Lees M., Sloane Stanley G.H. (1957). A simple method for the isolation and purification of total lipides from animal tissues. J. Biol. Chem..

[40] Hodson L., Skeaff C.M., Fielding B.A. (2008). Fatty acid composition of adipose tissue and blood in humans and its use as a biomarker of dietary intake. Prog. Lipid Res..

[41] Bjermo H., Risérus U. (2010). Role of hepatic desaturases in obesity-related metabolic disorders. Curr. Opin. Clin. Nutr. Metab. Care.

[42] Lönnroth P., Digirolamo M., Krotkiewski M., Smith U. (1983). Insulin binding and responsiveness in fat cells from patients with reduced glucose tolerance and type ii diabetes. Diabetes.

[43] Arner P., Bolinder J., Engfeldt P., Hellmér J., Ostman J. (1984). Influence of obesity on the antilipolytic effect of insulin in isolated human fat cells obtained before and after glucose ingestion. J. Clin. Investig..

[44] Paolisso G., Tataranni P.A., Foley J.E., Bogardus C., Howard B.V., Ravussin E. (1995). A high concentration of fasting plasma non-esterified fatty acids is a risk factor for the development of niddm. Diabetologia.

[45] Frigolet M.E., Gutierrez-Aguilar R. (2017). The role of the novel lipokine palmitoleic acid in health and disease. Adv. Nutr..

[46] Hodson L., Karpe F. (2013). Is there something special about palmitoleate?. Curr. Opin. Clin. Nutr. Metab. Care.

[47] Gong J., Campos H., McGarvey S., Wu Z., Goldberg R., Baylin A. (2011). Adipose tissue palmitoleic acid and obesity in humans: Does it behave as a lipokine?. Am. J. Clin. Nutr..

[48] Scagliusi F.B., Ferriolli E., Pfrimer K., Laureano C., Cunha C.S., Gualano B., Lourenco B.H., Lancha A.H. (2008). Underreporting of energy intake in brazilian women varies according to dietary assessment: A cross-sectional study using doubly labeled water. J. Am. Diet. Assoc..

[49] Mozaffarian D., Cao H., King I.B., Lemaitre R.N., Song X., Siscovick D.S., Hotamisligil G.S. (2010). Circulating palmitoleic acid and risk of metabolic abnormalities and new-onset diabetes. Am. J. Clin. Nutr..

[50] Warensjö E., Ohrvall M., Vessby B. (2006). Fatty acid composition and estimated desaturase activities are associated with obesity and lifestyle variables in men and women. Nutr. Metab. Cardiovasc. Dis..

[51] Okada T., Furuhashi N., Kuromori Y., Miyashita M., Iwata F., Harada K. (2005). Plasma palmitoleic acid content and obesity in children. Am. J. Clin. Nutr..

[52] Wang X., Lin H., Gu Y. (2012). Multiple roles of dihomo-gamma-linolenic acid against proliferation diseases. Lipids Health Dis..

[53] Aschner P., Buendía R., Brajkovich I., Gonzalez A., Figueredo R., Juarez X.E., Uriza F., Gomez A.M., Ponte C.I. (2011). Determination of the cutoff point for waist circumference that establishes the presence of abdominal obesity in latin american men and women. Diabetes Res. Clin. Pract..

[54] Haffner S.M., Miettinen H., Stern M.P. (1997). The homeostasis model in the san antonio heart study. Diabetes Care.

[55] Hanley A.J., Williams K., Stern M.P., Haffner S.M. (2002). Homeostasis model assessment of insulin resistance in relation to the incidence of cardiovascular disease: The san antonio heart study. Diabetes Care.

